# Short courses

**DOI:** 10.1155/S1463924689000209

**Published:** 1989


experiments were performed at room temperature. After injection, the mixture was led through the enzyme coil cell where the enzymatic reactions took place (figure 2), through a flow cell and from there to waste. The retention time was about 3 min.

The sample concentration was calculated on the basis of the peak height, by reference to a calibration graph obtained by linear regression.

### Results and discussion

Calibration data for glucose standards were measured at concentrations 0·00, 0·56, 1·11, 1·67, 2·78 and 3·33 mM with six determinations at each level. The correlation coefficient was 0·994 and the mean coefficient of variation (CV) was 1·5%, ranging from 0·0 to 3·6%. Carry-over was tested as described by Andersen and Hannibal [3] and was found to be 2%. The detection limit was found to be 0·10 mM, which covers the normal range of plasma glucose (4·2–6·7 mM) and hypo and hyperglycaemic levels resulting from metabolic disorders. The mean glucose concentration from a serum standard was found to be 6·3 mM (CV 2·75%, $n = 6$), compared with the declared concentration of 6·03–6·49 mM. When the glucose level was determined in serum or plasma, no interference of proteins was observed. The rate of determinations was 20 per hour because of the high viscosity, but dilution (3 : 5) with 70% ethanol increased the capacity to 40 determinations per hour. When full blood samples are analysed, addition of heparin (15 µl for a ca 200-µl sample) is needed, followed by centrifugation.

In conclusion, the system is simple and practical when many analyses are to be performed periodically.

### Acknowledgements

This work was supported by NOVO Industry A/S, Bagsvaerd, Denmark. The author thanks Mrs Lene Lykke Rasmussen for skilful technical assistance. Ib Andersen, Department of Clinical Chemistry, Copenhagen Country Hospital, Herlev, Denmark, is thanked for fruitful discussions and Arne Jensen, Department of Pharmaceutical Chemistry, Royal Danish School of Pharmacy, is thanked for providing the pump and all tubing.

### References

1. Hansen, E. H., in *Flow Injection Analysis* (Polyteknisk Forlag, Lyngby, Denmark, 1986), p. 66.
2. *Multichannel Biochemical Analyzers*, Technicon Method No. SF4-0046FA8 (Technicon Instruments, Tarrytown, NY, 1978).
3. Anderson, I. and Hannibal, S., *Journal of Automatic Chemistry*, **5** (1983), 188.

### Short courses

Loughborough University of Technology, UK, has announced the following short courses for 1989:

Fluorescence and Luminescence Spectrometry – 26–30 June 1989. Fee £480 including residence and all meals (£450 if paid with booking form). Non-residents £405 (£375).

Statistics for Analytical Chemistry – 11–14 July 1989. Fee £385 including residence and all meals (£355 if paid with booking form). Non-residents £325 (£295).

Flow Injection Analysis – 12–14 July 1989. Fee £325 including residence and all meals (£300 if paid with booking form). Non-residents £275 (£250).

*For further details please contact: Mrs J. E. Stirling, Department of Chemistry, Loughborough University of Technology, Loughborough, Leics. LE11 3TU. Telephone: (0509) 222549.*

HPLC Technology and Applied Chromatography Systems are holding five HPLC Beginners Training Courses during 1989. Each course will last three days and will include both practical and discussion sessions. The courses are held at The Deanwater Hotel, Woodford, Cheshire. All purchasers of HPLC Systems from ACS receive a complimentary place on the course.

*For further information please contact: Applied Chromatography Systems, The Arsenal, Heapy Street, Macclesfield, Cheshire SK11 7JB. Telephone: (0625) 34575.*

Chemserve have announced two courses for 1989:

Fluorine Spectroscopy Workshop – 10–11 April 1989.

Mass Spectrometry for Beginners – 17–18 April 1989.

*For further information, please contact: Chemserve, UMIST, PO Box 88, Manchester M60 1QD. Telephone: 061 228 7700.*

The University of Liverpool is holding a short course on Modern Spectroscopic Techniques, 2–7 April 1989.

*For further information, please contact: Dr A. Hodgson, Dept. of Chemistry, University of Liverpool, PO Box 147, Liverpool L69 3BX.*

Royal Society of Chemistry Residential School – 28–31 March 1989. Computer Methods in UV, vis and ir Spectroscopy, Polytechnic of Wales.

*For further information, contact Ms L. Hart, RSC, 30 Russell Square, London WC1B 5DT. Telephone: 01-631-1355.*

